# Population genomics for wildlife conservation and management

**DOI:** 10.1111/mec.15720

**Published:** 2020-11-18

**Authors:** Paul A. Hohenlohe, W. Chris Funk, Om P. Rajora

**Affiliations:** ^1^ Department of Biological Sciences and Institute for Bioinformatics and Evolutionary Studies University of Idaho Moscow Idaho USA; ^2^ Department of Biology Graduate Degree Program in Ecology Colorado State University Fort Collins Colorado USA; ^3^ Faculty of Forestry and Environmental Management University of New Brunswick Fredericton New Brunswick Canada

**Keywords:** adaptive capacity, conservation units, effective population size, genetic rescue, inbreeding depression, population connectivity

## Abstract

Biodiversity is under threat worldwide. Over the past decade, the field of population genomics has developed across nonmodel organisms, and the results of this research have begun to be applied in conservation and management of wildlife species. Genomics tools can provide precise estimates of basic features of wildlife populations, such as effective population size, inbreeding, demographic history and population structure, that are critical for conservation efforts. Moreover, population genomics studies can identify particular genetic loci and variants responsible for inbreeding depression or adaptation to changing environments, allowing for conservation efforts to estimate the capacity of populations to evolve and adapt in response to environmental change and to manage for adaptive variation. While connections from basic research to applied wildlife conservation have been slow to develop, these connections are increasingly strengthening. Here we review the primary areas in which population genomics approaches can be applied to wildlife conservation and management, highlight examples of how they have been used, and provide recommendations for building on the progress that has been made in this field.

## 1 INTRODUCTION

### 1.1 The need for population genomics in wildlife biology

As increasing attention is focused on global change and loss of biodiversity (IPBES, 2019), it is critical to understand the changes and challenges that wildlife populations face and use the tools now available for management and conservation of wildlife species. Central issues in wildlife conservation include identifying populations and units for conservation, assessing population size and connectivity, detecting hybridization, assessing the potential of populations to persist and adapt to environmental change, and understanding the factors that affect this potential. Genetic information can inform all of these issues and provide critical information for designing management strategies to address them. The genomics revolution has democratized the field of population genomics, allowing high‐throughput sequencing to be applied in nearly any organism, including natural populations of rare or difficult‐to‐study species (Supple & Shapiro, 2018; Luikart et al., 2019; Rajora, 2019). As a result, genomics approaches are an important part of the toolkit for a basic understanding of wildlife biology, such as disease or population dynamics, and to inform direct conservation and management actions for wildlife populations and their habitats. At the same time, the power of genomics techniques presents new challenges for researchers in analysing and interpreting large genomic data sets.

Natural populations face a number of threats, including habitat loss and alteration, direct mortality from exploitation, invasive species, emerging infectious disease, pollution and climate change. These threats are pervasive and global, so that an estimated 1 million species of plants and animals are at risk of extinction within the next few decades (IPBES, 2019). Threats to wildlife populations often act synergistically, and genetic factors are central to the challenges confronting wildlife. For instance, loss of genetic diversity and inbreeding due to population declines and fragmentation can reduce population fitness directly, but also can reduce a population's ability to adapt to novel conditions produced by invasive species or climate change (Ceballos et al., 2017). Genetics and genomics concepts, and the ability to efficiently study genetic factors in nature, are important for quantifying and mitigating threats to wildlife populations.

Several years ago, spurred by technological advances in high‐throughput sequencing, a set of reviews and perspective articles assessed the potential for the field of conservation genomics (e.g., Allendorf et al., 2010; Primmer, 2009; Steiner et al., 2013). Genomics concepts and approaches have a wide range of applications in conservation, from seed sourcing for restoration to understanding community‐level effects of genomic diversity (Breed et al., 2019; Hand et al., 2015; Holliday et al., 2017; Rajora, 2019). Here we focus on applications of population genomics to wildlife, which we define as natural populations of terrestrial vertebrate species that are the focus of specific attention for conservation or population management (although most of the tools and concepts we discuss are applicable to all of biodiversity, and in particular wildlife biology can learn from applications of population genomics in fisheries). Over the last decade, the field has made substantial progress in understanding how to apply population genomics in wildlife and what questions can be addressed. It is timely to take stock of the progress that has been made to date, learn from some of the successes, and identify avenues for future progress in wildlife population genomics research. Additionally, a critical need is to translate wildlife population genomics research to conservation actions, requiring concrete steps toward integrating the two.

### 1.2 Approaches in population genomics

Traditional conservation genetics in wildlife has relied on techniques including allozyme and microsatellite genotyping or sequencing of mitochondrial DNA to provide a wealth of knowledge about natural populations (Allendorf, 2017). However, these techniques provide data on a limited number of genetic markers across individuals. Advances in next‐generation sequencing technology have led to a proliferation of techniques for population genomics studies, all of which have the potential to provide fine‐scale genetic data across the genome of multiple individuals (Holliday et al., 2019). Multiple genomics techniques provide sequence data on a reduced representation of the genome, such as the transcriptome or a pre selected set of loci targeted with primers or hybridization probes (Meek & Larsen, 2019). Anonymous reduced‐representation techniques provide sequence data from loci spread across all parts of the genome, which are determined by the molecular protocol, such as the choice of restriction enzymes used in the restriction‐site associated DNA sequencing (RADseq) family of techniques (Andrews et al., 2016). Finally, whole‐genome sequencing (WGS) produces data from every part of the genome, and it is increasingly feasible for most taxa (Fuentes‐Pardo & Ruzzante, 2017). Importantly for studies of wildlife species, many of these techniques, including transcriptome, RADseq and WGS, do not require any prior genomic knowledge for the species.

The line between genetics and genomics, and whether it is even useful to make a distinction, is subject to differing opinions. The vast increase in the amount of data provided by genomics techniques can allow new questions to be addressed, such as detection of genes associated with important traits or fitness, that were not tractable with traditional techniques; this has been called “narrow‐sense genomics” (Garner et al., 2016; Hohenlohe, Hand, et al. 2019). With the availability of reference genome assemblies, placing genetic markers on chromosomes provides important information about physical linkage and recombination and connects genetic markers directly to candidate genes. This new perspective can be integral to a truly genomics study, and what Allendorf (2017) calls “the death of beanbag genetics.” Conversely, in a “broad‐sense genomics” approach (Garner et al., 2016), high‐throughput sequencing tools can be used to address questions that were already tractable with traditional genetic techniques. The advantage of using newer techniques is increased statistical power and resolution with more markers, and in many cases increased efficiency and cost‐effectiveness (Walters & Schwartz, 2020).

### 1.3 Applications to wildlife

Below we highlight a number of recent applications of population genomics to understanding wildlife populations. Progress in this field has revealed several general trends. First, all of the techniques described above, from traditional genetics tools through the wide range of next‐generation sequencing approaches, continue to have important roles to play (as predicted by Primmer, 2009). Determining which approach is best in a particular case depends on many factors, including the resources available and the data required to address a specific scientific question (Hohenlohe, Hand, et al. 2019). Second, population genomics studies are increasingly able to address multiple scientific questions with high precision from a single genomic data set. For instance, genomic data can allow population structure to be assessed from the perspective of both neutral and adaptive connectivity, with different implications for conservation actions (Funk et al., 2012). WGS data from a relatively small number of individuals can provide information across a range of timescales, from demographic history and phylogenetic relationships among widely separated populations over the last two million years, to inbreeding within the last century (Saremi et al., 2019). In part this is the result of new analytical approaches made possible by genomic data sets, such as demographic reconstruction (discussed below) and runs of homozygosity (ROH; Box 1).

Box 1 **Understanding Inbreeding: runs of homozygosity**

Loss of genetic diversity and inbreeding in small populations is a central threat to many wildlife populations. With fine‐scale genomic data, such as short‐read WGS data, mapped to a reference genome, it is possible to identify runs of homozygosity (ROH) – chromosomal regions that have few or no heterozygous nucleotide sites because both chromosome copies derive from a single copy in a relatively recent common ancestor (Ceballos et al., 2018). The proportion of the genome that is in ROH, or identical by descent, has long been central to the concept of inbreeding, because it is the result of relatedness between parents. Being able to map these regions in the genome reveals several novel insights that illustrate the power of population genomics approaches. First, ROH provide precise estimates of individual‐level inbreeding which are more accurate than other methods (Kardos et al., 2015).

Further, the lengths of ROH reveal details of demographic history and the timescale of inbreeding (Grossen et al., 2020). Part a of the figure shows average heterozygosity across the genome of several wolf (*Canis* spp.) individuals from Robinson et al. (2019); regions where heterozygosity is absent are ROH. The individual from and outbred population in Minnesota shows relatively high heterozygosity and very few ROH. The individual from Ethiopia had low genome‐wide heterozygosity due to long‐term small effective population size in an isolated population, but few long ROH suggesting relatively little contemporary inbreeding. In contrast, the individual from the severely declining (now extinct) population on Isle Royale, Michigan, USA, shows several long ROH across the genome, as expected with recent inbreeding. Because recombination breaks up haplotype blocks with each generation, smaller ROH reflect older inbreeding events, so that the distribution of ROH lengths tells the history of inbreeding in a population. For example, part b of the figure shows the distribution of ROH lengths in 10 puma (*Felis concolor*) individuals. Size classes of ROH correspond to the expected number of generations since the individual’s maternal and paternal lineages shared a common ancestor for that chromosomal region (Saremi et al. 2019).

Genes that cause inbreeding depression due to recessive deleterious alleles in the homozygous state or the loss of heterozygosity at particular genes can be mapped by comparing the locations of ROH across individuals. Further, the relative locations of ROH among individuals and populations can be informative for controlled breeding or genetic rescue attempts. For example, if two individuals share ROH at the same chromosomal region due to common ancestry, their offspring will also have those regions of reduced diversity. However, if two individuals have different ROH, mating between them can produce offspring with lower inbreeding coefficients, potentially relieving inbreeding depression. Part c shows the extent of ROH sharing among puma individuals (Saremi et al., 2019); many pairs show only minimal sharing of ROH, but two individuals from Florida (CYP47 and CYP51) share ROH across a relatively large portion of their genomes due to identity by descent from inbreeding, and any offspring from this pair would also be severely inbred.

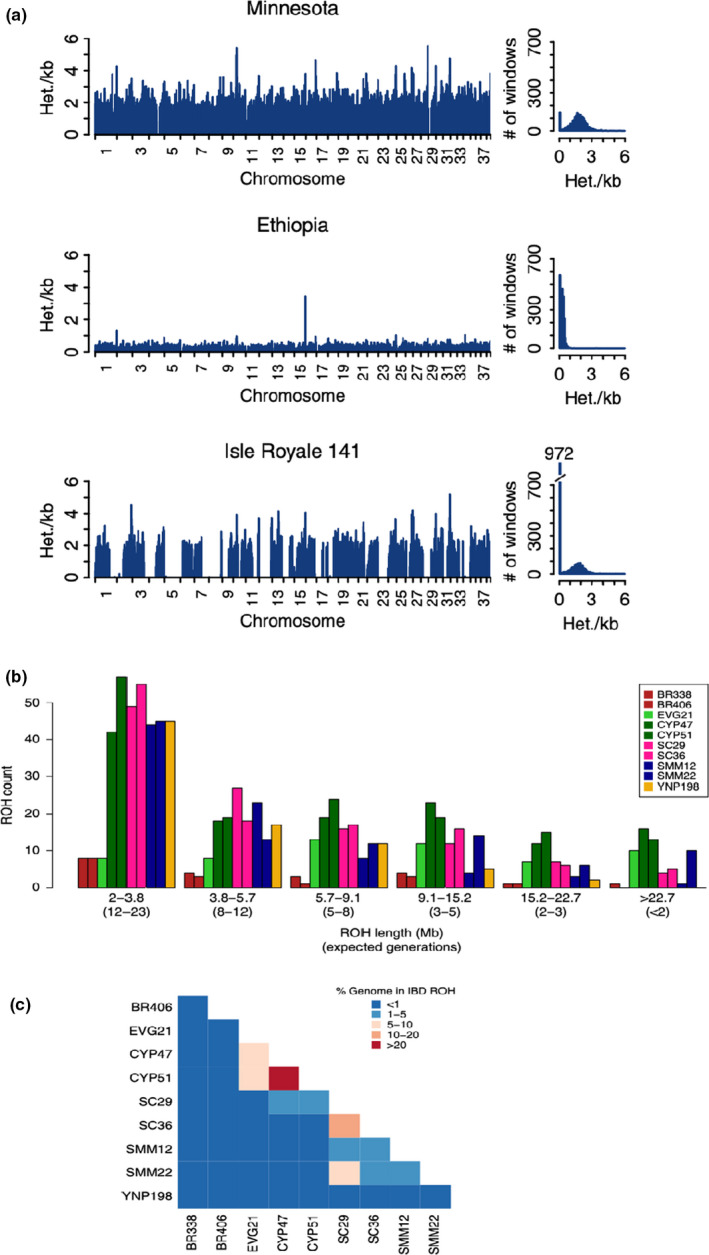

Third, many approaches that are most useful for wildlife also combine multiple population genetics or genomics approaches. For instance, many applications of genetics tools in wildlife require the ability to genotype a set of genetic markers consistently over time across many individuals, for instance in long‐term monitoring of populations. Next‐generation sequencing tools can efficiently provide a large amount of data, from which a highly optimized set of marker loci can be extracted for specific objectives such as parentage analysis, population assignment or monitoring of adaptive loci (Förster et al., 2018; Hess et al., 2015; Meek et al., 2016; von Thaden et al., 2020). These marker panels may have relatively few loci (e.g., orders of magnitude fewer than the genomic data set on which they are based) and miss large parts of the genome, so they may not be considered “genomics” in a strict sense. Nonetheless, when a selected panel of marker loci is developed from a genome‐wide data set to include adaptively significant loci, it is able to address questions about adaptive variation in wildlife populations that were previously intractable with traditional genetics studies.

A variety of other technical advances and available resources facilitate the use of population genomics in wildlife species. Increasingly, sequencing technology is advancing to the point that it can be used in the field with only a backpack full of equipment and supplies, for instance using MinION nanopore sequencing technology (Oxford Nanopore Technologies; Krehenwinkel, Pomerantz, & Prost, et al., 2019; Krehenwinkel, Pomerantz, Henderson, et al., 2019). Increasing numbers of wildlife species have reference genome assemblies available, and these provide a number of benefits, including improved identification of loci, linking genetic markers to candidate genes, and haplotype‐based or other analyses that are not possible otherwise (Brandies et al., 2019; Luikart et al., 2019; Box 1). If a reference genome is not available for a particular species, one from a closely related species can be used to align short‐read sequence data (e.g., Janecka et al., 2020 aligned WGS data from snow leopards against the tiger genome assembly, both in the genus *Panthera*), and it can also provide a backbone for creating a reference genome assembly for the focal species. The growing number of reference genome assemblies is facilitated by large collaborative initiatives focused on taxonomic groups, such as Australian mammals (https://ozmammalsgenomics.com), birds (Zhang et al., 2014; https://b10k.genomics.cn/index.html) or all eukaryotes (Lewin et al., 2018). Transcriptomic and epigenetic databases also provide complementary information, especially useful for genome annotation and gene functional insights.

### 1.4 Use of noninvasive and low‐quality DNA samples

A particular need in wildlife studies is the ability to use low‐quality and/or low‐quantity DNA, including DNA extracted from archival or historical samples, noninvasive samples from hair, feathers or faeces, and environmental DNA (eDNA) from water or other environmental samples. Although some genomics techniques such as WGS require DNA samples of relatively high concentration or molecular weight, a growing range of techniques can be applied to low‐quality DNA samples (Andrews et al., 2016; Andrews, deBarba, et al., 2018). In general, these methods target fewer loci than other approaches and may be particularly useful for monitoring (Carroll et al., 2018). Targeted sequencing approaches, using primers for amplification or hybridization probes, are particularly effective (Bi et al., 2019; Schmidt et al., 2020), and White et al. (2019) provide detailed information on optimizing capture approaches using faecal samples from chimpanzees. These methods can be applied to fragmented DNA samples because they target relatively small chromosomal regions (e.g., sometimes <100 bp) but the trade‐off is that these loci must be identified from previous sequence data. Other methods, such as RADseq and WGS, have also seen progress in application to low‐quality samples (Andrews, deBarba, et al., 2018). For instance, Chiou and Bergey (2018) present a methylation‐based method that enriches vertebrate DNA relative to bacterial DNA from faecal samples as an initial step, allowing for approaches such as RADseq. Conversely, sequencing focused on the microbial genomes of faecal samples, or other microbiome samples, can also provide useful information in wildlife studies (West et al., 2019).

In difficult‐to‐study species, it can be useful to combine genotyping of noninvasive samples at traditional markers such as microsatellites with genomic sequencing of a few individuals, such as captive individuals, for which higher quality DNA samples are available (for instance in snow leopards, *Panthera uncia*; Janecka et al., 2020). Panels of single‐nucleotide polymorphisms (SNPs) optimized from large genomic data sets can also be genotyped using low‐quality DNA samples (Andrews, deBarba, et al., 2018; von Thaden et al., 2020). Particularly in threatened wildlife species in which genetic variation has been lost in living populations but remains in archival museum or field‐collected ancient samples, techniques for analysing low‐quality DNA samples open a window into the genetic past that can inform current conservation efforts (Bi et al., 2013; van der Valk, Díez‐del‐Molino, et al., 2019). Techniques for low‐quality samples are also important for wildlife forensics; for instance, Natesh et al. (2019) tested amplicon sequencing methods in degraded tiger samples and even in cooked queen conch samples as a method to confirm species identity and even source population.

Sequencing of eDNA has primarily been used for detection of species presence in aquatic ecosystems (e.g., Marshall & Stepien, 2019). However, it has been applied to terrestrial wildlife species, for instance by sampling from footprints in snow (Franklin et al., 2019; Kinoshita et al., 2019). Use of eDNA for truly population genetic studies (e.g., to estimate allele frequencies) is challenging in aquatic systems because DNA fragments cannot be assigned to individuals (but see Sigsgaard et al., 2017), but terrestrial samples such as footprints may alleviate this issue.

## 2 UNDERSTANDING WILDLIFE POPULATIONS

### 2.1 Population size and demographic history

Perhaps the most basic aspect of wildlife populations that can be addressed with population genomics tools is population size. The number of individuals is a key factor in determining demographic viability of populations and in determining management actions, such as harvest quotas based on numbers of adults, recruitment rates and knowledge of source sink dynamics. Genetics tools, such as marker panels designed for individual identification, can be used in genetic mark–recapture studies to estimate population densities, including noninvasive samples such as scat and hair (Mills et al., 2000; von Thaden et al., 2020). Genetic marker panels that are able to estimate close kinship relationships can similarly be used to estimate population size (Bravington et al., 2016; Clendenin et al., 2020). As described above, genomics tools can provide efficient methods for designing such marker panels from strict filtering of a much larger set of loci.

Population size is critical not only for demographic viability of wildlife populations, but also because of its effect on genetic diversity. This is captured by the effective population size (*N_e_*), defined as the size of an ideal, panmictic population that would experience the same loss of genetic variation, through genetic drift, as the observed population. *N_e_* is usually smaller than the observed “census” population size (*N_c_*), due to a number of factors common in natural populations, particularly wildlife taxa, including fluctuating population size, variance in reproductive success and overlapping generations, although there is wide variation in the *N_e_*/*N_c_* ratio (Charlesworth, 2009). *N_e_* influences the likelihood of accumulation of deleterious variants, inbreeding depression, and the capacity of populations to adapt to environmental change or disease, important factors in wildlife populations that are declining or have experienced bottlenecks.

**Figure 1:**
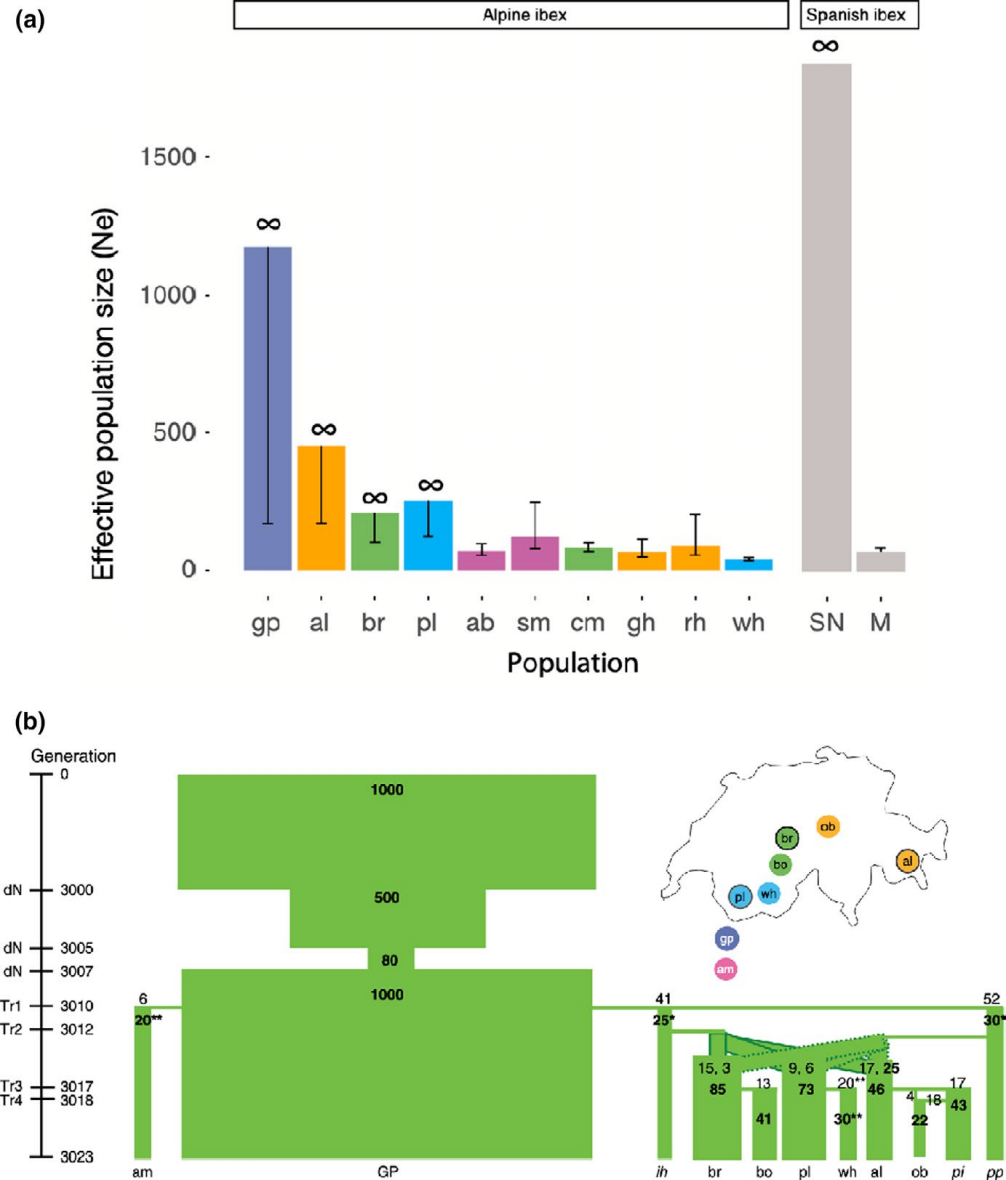
Two types of genomic data have been used to estimate population size and demographic history in Alpine ibex (*Capra ibex*). Several reintroduced populations in Switzerland were derived from the same Italian source population, Gran Paradiso (GP). Other populations are Albris (al), Brienzer (br), Pleureur (pl), Aletsch Bietschhorn (ab), Schwarz Mönch (sm), Cape au Moine (cm), Graue Hörner (gh), Rheinwald (rh), Weisshorn (wh), Sierra Nevada (SN), Maestrazgo (M), Zoo Interlaken Harder (ih), Bire Öschinen (bo), Oberbauenstock (ob), Pilatus (pi), Wildpark Peter and Paul (pp). (a) Contemporary estimates of *N_e_* across multiple populations of Alpine ibex and a related species based on RADseq‐derived SNP loci and analysis of linkage disequilibrium. Note that confidence limits, particularly the upper limit, can be large or even infinite. Reproduced from Grossen et al. (2018). (b) WGS data can provide estimates of current *N_e_* (shown as numbers in bold) as well as reconstruction of demographic history. Here time goes from top to bottom, and the width of the green bars corresponds to *N*_e_ within a time period. Generation 3,023 represents current populations. Reproduced from Grossen et al. (2020)

Population genomics approaches can be used to estimate *N_e_* (Browning & Browning, 2015; Kardos et al., 2017). For instance, Nunziata and Weisrock (2018) used simulations to test the potential for RADseq data sets to estimate *N_e_* and declines in *N_e_* over time, using methods based on linkage disequilibrium (LD) and the site frequency spectrum (SFS). They found that RADseq data are effective for precisely estimating *N_e_* and for detecting declines in *N_e_* over contemporary timescales (20 generations), and that LD‐based methods are superior, provided a sufficient sample size of individuals. If a reference genome assembly with data on recombination rate is available, methods to estimate *N*_e_ based on LD among linked loci may be even more effective (Hollenbeck et al., 2016; Lehnert et al., 2019). Grossen et al. (2018) used RADseq to generate >100,000 SNPs to test the genetic effects of reintroduction of Alpine ibex (*Capra ibex*) in Switzerland and found markedly reduced LD‐based estimates of *N_e_* in reintroduced populations compared to the source population or the closely related Iberian ibex (*C. pyrenaica*) (Figure 1a). Nunziata et al. (2017) also found that demographic model inference of changes in *N_e_* based on double digest RAD (ddRAD) data from two salamander species (*Ambystoma talpoideum* and *A. opacum*) agreed with population size changes inferred from mark–recapture data; because this study included ddRAD sequencing on samples collected decades ago, temporal trends in *N_e_* could be estimated for these two species using both mark–recapture and ddRAD. Jensen et al. (2018) compared variation at >2,000 SNPs in Pinzón giant tortoise (*Chelonoidis duncanensis*) samples from a single island in the Galápagos Island from before and after a bottleneck that reduced *N_e_* to just 150–200 in the mid‐20th century. They found that the extent and distribution of genetic variation in the historical and contemporary samples was very similar, which they attributed to a successful ex situ head‐start and release programme.

Even in the absence of historical samples, population genomic data can be used to uncover the demographic history of populations, including population bottlenecks and expansions. Because loss of genetic diversity and consequences for population fitness depend strongly on not only the severity but also the timescale of population bottlenecks, reconstructing demographic history in wildlife species can help explain current levels of genetic diversity. While historical trends can be estimated from large SNP data sets, WGS from a few individuals is effective in producing demographic reconstructions. In this case, conclusions rely on the assumption that the individuals sequenced are truly representative of the population under study, and inference from one or a few individuals does include some unavoidable sampling variance (King et al., 2018). Estimates of *N*_e_ are also affected by historical population structure and migration (Mazet et al., 2016). Methods include the sequentially Markovian coalescent (SMC; Li & Durbin, 2011; Terhorst et al., 2017) or the site frequency spectrum (SFS; Liu & Fu, 2015); SMC may better detect older population fluctuations, and SFS more recent ones (Patton et al., 2019). This approach has provided additional insights into the Alpine ibex case, suggesting that despite a dramatic demographic recovery, Alpine ibex carry a persistent genomic signature of their reintroduction history (Grossen et al., 2020; Figure 1b; Box 1). Demographic analyses by Ekblom et al. (2018) using WGS of 10 Scandinavian wolverines (*Gulo gulo*) uncovered a long‐term decline of the population from an *N_e_* of 10,000 well before the last glaciation to <500 after this period, indicating that this population has been declining for thousands of years. Two subspecies of gorilla also provide an illustrative contrast: in Graur's gorilla (*Gorilla beringei graueri*), population declines have led to loss of genetic diversity and increased inbreeding, while the mountain gorilla (*G. beringei beringei*) population has remained small but genetically stable over the past century (van der Valk et al., 2019). This study was enabled by WGS of both museum and contemporary samples. Historical demographic reconstruction can link population changes to environmental shifts, with the potential to predict the effect of ongoing environmental changes on population distributions and genetic diversity (Prates et al., 2016).

Low genetic variation and small *N_e_* do not necessarily mean that a population will suffer from inbreeding depression. Genetic load, the negative consequences of deleterious variation that can accumulate from genetic drift, may be purged in small populations, and some populations appear to experience few negative fitness effects despite low genetic variation. Testing for inbreeding depression requires combining genetic data with fitness data or delving deeper into the function of alleles prevalent in small populations due to genetic drift. One approach for assessing the potential for inbreeding depression is to predict the physiological and fitness consequences of specific allelic variants at high frequency or fixed in small, inbred populations (e.g., Grossen et al., 2020). Benazzo et al. (2017) found several private and deleterious amino acid changes fixed due to genetic drift in Apennine brown bears (*Ursus arctos marsicanus*) that are predicted to result in energy deficit, muscle weakness, skeletal and cranial anomalies, and reduced aggressiveness. Arguably the strongest evidence for inbreeding depression comes from studies that show a negative correlation between fitness and inbreeding coefficients. Huisman et al. (2016) found strong evidence for inbreeding depression in red deer (*Cervus elaphus*) by examining the relationship between several different fitness metrics and inbreeding coefficients estimated using SNPs. In contrast, inbreeding coefficients calculated from a deep and fairly complete pedigree in the same population found evidence for inbreeding depression for fewer traits (Huisman et al., 2016), highlighting the emerging consensus that genomic estimates are better for quantifying inbreeding than pedigrees (Kardos, Taylor, et al., 2016). Estimates of ROH, especially from WGS data, are particularly effective at both quantifying inbreeding coefficients and understanding candidate loci underlying inbreeding depression (Box 1).

### 2.2 Population structure and connectivity

A long‐standing goal of population genetics, and critical source of information for conservation and management actions in wildlife, is to identify populations and understand the relationships among them. Characterizing population structure, the distribution of genetic variation within and among populations, is key for inferring the relative importance of different evolutionary processes (gene flow, drift and selection) across populations. Given that gene flow infuses new genetic variation into populations, there is also a strong interest in wildlife and conservation biology in understanding the amount of gene flow among populations, particularly those isolated in fragmented landscapes (Crooks and Sanjayan, 2006; Walters & Schwartz, 2020).

**Figure 2:**
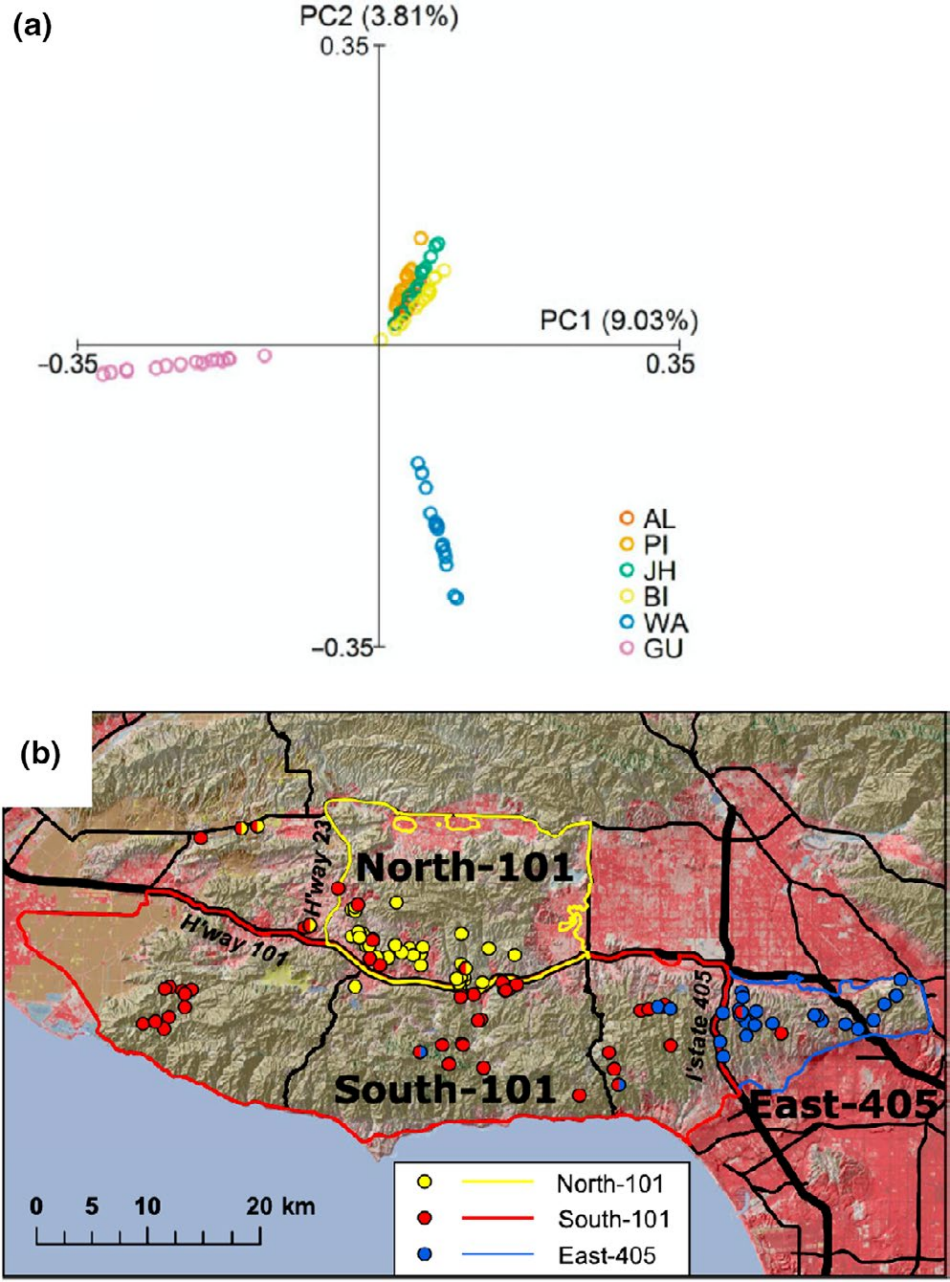
Inferring population structure in wildlife species. (a) Principal Components Analysis based on WGS reveals genetically differentiated populations of sage‐grouse. The Gunnison sage‐grouse (GU; *Centrocercus minimus*) had previously been recognized as a separate species, while the genetic distinctiveness of the Washington population (WA) of greater sage‐grouse (*C. urophasianus*) from all other populations of this species was revealed by this study. Reproduced from Oh et al. (2019). (b) Genomic analysis of bobcat (*Lynx rufus*) populations in southern California showing the effect of major highway corridors on gene flow. Coloured points represent individuals assigned to genetic population groups, and red and black lines represent major highways. Reproduced from Kozakiewicz et al. (2019)

The first step in inferring population structure using genetic or genomic data is to delineate populations. What constitutes a population is not always obvious for natural populations, and it is important to distinguish demographic and genetic connectivity (Lowe & Allendorf, 2010; Waples & Gaggiotti, 2006). This is particularly true for continuously distributed populations, but also for species distributed in discrete habitat patches, which may or may not be equivalent to populations (Funk et al., 2005). Fortunately, population genomics provides increased power to delineate populations, detect cryptic population structure and quantify how genetically different populations are. For example, Oh et al. (2019) identified a genetically very divergent population of greater sage‐grouse (*Centrocercus urophasianus*) in eastern Washington state using WGS of representative individuals, which has important implications for conservation of this imperiled species (Figure 2a). The scale of genomic data also allowed the researchers to link population structure to adaptive divergence at candidate loci associated with detoxification of the birds’ primary food, sagebrush (*Artemisia* spp.). In another example, mitogenomic (Hofman et al., 2015) and RADseq‐generated SNP data (Funk et al., 2016) revealed evidence for a low level of historical gene flow in island foxes (*Urocyon littoralis*) among island populations, which suggests recent human movement of foxes. In these examples, genetic and genomic data confirmed the expected delineation of populations by geography, but also quantified the differentiation among them.

In other cases, geographical delineation of populations is not as clear. Landscape genetics combines population genetics, landscape ecology and spatial statistics to understand the effects of landscape and environmental heterogeneity on gene flow, genetic variation and microevolutionary processes, and to identify barriers between populations (Manel et al., 2003). Genomics tools add statistical power and resolution to these studies, and also add the potential to identify loci associated with adaptation within and among populations. This has led to the distinction between neutral landscape genomics (addressing the questions of traditional landscape genetics with genomics tools) and adaptive landscape genomics (Forester et al., 2018; Storfer et al., 2018); we discuss genomics studies of adaptive genetic variation below. One advantage of landscape genetics and genomics is that the unit of analysis can be either the individual or the population, which facilitates studies of organisms that are continuously distributed, rather than clustered in discrete patches. A focus of landscape genetics and genomics studies of wildlife species has been to understand how anthropogenic habitat modification influences patterns and rates of gene flow. For instance, Kozakiewicz et al. (2019) found that urbanization impedes connectivity among bobcat (*Lynx rufus*) populations in southern California, and the barrier effect of major highway corridors can be seen in the genetic separation of wildlife populations (Figure 2b). Genomic data can also reconstruct the historical patterns of gene flow among populations, whether natural or human‐mediated (Figure 1b), and link these to the geographical and climatic factors causing changes in gene flow over time. This puts contemporary patterns of genetic variation and reductions in connectivity due to habitat fragmentation in a historical context. As an example, Hotaling et al. (2018) analysed SNPs generated using RADseq with coalescent‐based demographic modelling to investigate historical patterns of gene flow in a rare, stream stonefly (*Lednia tumana*) in the Rocky Mountains of Glacier National Park, Montana, USA. Their analyses supported divergence with gene flow among three genetic clusters since the end of the Pleistocene (~13,000–17,000 years ago), which they interpreted as the result of south‐to‐north recession of ice sheets.

### 2.3 Hybridization and admixture

An emerging view in evolutionary biology in the last few decades is that hybridization between animal species is relatively common and plays an important role in evolution and ecology. For instance, Toews et al., (2019) reviewed the evidence that admixture between bird species has been an important source of variation and has possibly led to the formation of new species. Population genomic approaches can provide large sets of markers that increase the ability to detect and quantify low levels of hybridization or admixture (the flow of genetic variation into a species or population as a result of hybridization) (Luikart et al., 2019). Large SNP data sets can estimate historical hybridization events among related taxa, using methods that rely on shared allelic variation across a phylogeny (e.g., Foote & Morin, 2016; Sinding et al., 2018). Additionally, mapping genomic data onto a reference genome assembly can identify chromosomal tracts of ancestry. Because these blocks of ancestry break down through recombination following a hybridization event, the distribution of their sizes can be used to infer the history of hybridization and admixture in wildlife species, as well as evidence for selection in admixed genomes (e.g., Leitwein et al., 2018, 2019).

Admixture can have both negative and positive effects on population fitness. In snowshoe hares (*Lepus americanus*), Jones et al. (2018) found that brown winter coats probably originated from an introgressed black‐tailed jackrabbit (*L. californicus*) allele that has swept to high frequency in parts of the snowshoe hare range with milder winter climates. Adaptive introgression into this species may have allowed it to expand its range following Pleistocene glaciation (Jones et al., 2020), and this genetic variation may play a key role in future adaptation as snowshoe hares encounter reduced winter snow cover across more of their range. Hybridization and admixture can also have negative consequences for fitness and local adaptation in wildlife species, particularly with massive increases in human‐facilitated movement of organisms (Allendorf et al., 2001). One example is species invasions facilitated by hybridization (e.g., feral swine, *Sus scrofa*; Smyser et al., 2020), which can negatively impact native wildlife populations. More directly, hybridization between westslope cutthroat trout (*Oncorhynchus clarkii lewisi*) and the widely introduced rainbow trout (*O. mykiss*) in western North America reduces fitness of the native species (Muhlfeld et al., 2009). Muhlfeld et al. (2017) amassed an impressive, multidecadal data set consisting of >12,000 individuals from 582 sites genotyped at allozyme loci, microsatellite loci and SNPs to infer the spatiotemporal dynamics of hybridization between these two species. They found that hybridization was more common in close proximity to historical stocking locations for rainbow trout, in warm water and with lower spring precipitation. Importantly, cold sites were not protected from invasion, meaning that even cutthroat trout populations in high‐elevation, cold water streams are not safe from hybridization by invasive rainbow trout. Large population genomic data sets will have greater power to detect and quantify even low rates of hybridization.

Identifying hybrids is also important from a legal standpoint, as hybrids between endangered and nonendangered species may not be protected under some endangered species laws (vonHoldt et al., 2017). The red wolf (*Canis rufus*) is listed as endangered under the U.S. Endangered Species Act (ESA), but recent hybridization with coyotes (*Canis latrans*) as well as historical hybridization with coyotes and other wolf taxa has resulted in substantial controversy. Nonetheless, Waples et al. (2018) found that under any historical pattern of hybridization, red wolves retain the basic features necessary to be considered a distinct population segment under the law and thus are eligible to remain on the list. Another North American canid species, eastern wolves (*Canis lycaon*), also has a complex history including recent hybridization. Heppenheimer et al. (2019) argue that such admixed populations still retain genetic variation representative of a distinct taxon and potentially important for local adaptation, warranting their protection under wildlife conservation measures.

## 3 ADAPTIVE VARIATION

### 3.1 The role of adaptive variation in wildlife

Determining the genetic basis of adaptive traits has been a central goal in evolutionary biology since the genesis of the field but has proved elusive for nonmodel species, such as wildlife. Historically, testing for local adaptation and dissecting its genetic basis required controlled breeding, common garden and reciprocal transplant experiments, which are typically only feasible for some model plant and animal species. As predicted by Allendorf et al. (2010), Steiner et al. (2013) and others, population genomics approaches have been widely used in recent years to assess adaptive genetic variation in natural populations, with implications for conservation and management. Adaptive variation in wildlife populations determines their long‐term viability, potential for increases in distribution or population size, and extinction probability. Wildlife populations face a variety of threats, including climate change and other factors that can be projected into the future. The quick pace of environmental change means that sensitive species will have to move, acclimatize or respond plastically, or evolve to avoid extinction (Dawson et al., 2011), but conservation actions can be targeted to facilitate these processes if they can be based on data about the genetic basis of adaptive variation. Additionally, some laws designed to protect endangered wildlife such as the U.S. ESA take adaptive potential into consideration in endangered species listing and delisting decisions (Funk et al., 2019).

Basic estimates of heritability of potentially adaptive traits can be informative. For instance, Reed et al. (2011) developed an individual‐based model to explore potential evolutionary changes in migration timing and the consequences for population persistence in Fraser River sockeye salmon (*Oncorhynchus nerka*). Assuming a heritability of migration timing of 0.5, they predict that adult migration timing will advance by ~10 days in response to a 2°C increase in temperature and that quasi‐extinction risk will only be 17% of that faced by populations with no evolutionary potential. Many wildlife species that are the focus of long‐term studies have pedigree data that can be used to estimate heritability of phenotypic traits (e.g., de Villemereuil et al., 2019), and genomics tools can also be used in natural populations to provide estimates of heritability by providing pairwise estimates of individual relatedness (Gienapp et al., 2017). Beyond assessing whether adaptive phenotypic traits have a genetic basis, population genomics now makes it possible to pinpoint the specific genes underlying this variation in natural populations, and better understand the processes and potential for adaptation. A genomic understanding of adaptive potential allows future projections of population viability and distribution under alternative scenarios of environmental change (Box 2).

Box 2 **Adaptive potential**

Adaptive potential (also called evolutionary potential) is the ability of a population to evolve genetically based changes in traits in response to changing environmental conditions (Funk et al., 2019). This is a component of the broader concept of adaptive capacity, which also includes nongenetic responses to environmental change, such as phenotypic plasticity and dispersal (Dawson et al., 2011; Nicotra et al., 2015). Species or populations with high adaptive potential are thus predicted to be less vulnerable to environmental change and more likely to survive in parts of their current distribution. Currently, we have a poor understanding of adaptive potential in many wild populations, so we do not know the extent to which it can buffer populations from rapid environmental change.

Adaptive potential depends on genetic variation in resilience traits among individuals within populations, as well as genetic differences in these traits among populations and across environmental gradients. Population genomics provides methods for estimating the genetic variation or heritability of traits that are expected to be important for adaptation, or for fitness per se. de Villemereuil et al. (2019) assessed adaptive potential in the hihi (*Notiomystis cincta*), an endangered New Zealand passerine (Chen, 2019). Combining RADseq and long‐term phenotypic and fitness data, they found a lack of genome‐wide diversity, low heritability of traits under selection, and little additive genetic variance of fitness, all indicating low adaptive potential in the sole remaining natural population and in a reintroduced population. Genomic evidence for a response to selection under current environmental stressors can reveal genetic variation and adaptive potential, for example in the case of disease such as transmissible cancer in Tasmanian devils (Epstein et al., 2016) or white‐nose syndrome in bats (Auteri and Knowles, 2020).

Another approach for assessing adaptive potential, particularly in the face of climate change, is to examine patterns of local adaptation to climate conditions across the current species range, and then project future climatic changes and species’ responses (e.g., Prates et al., 2016; Ruegg et al., 2018; Waterhouse et al., 2018). Adaptive differences among populations can contribute to adaptive potential and can also inform assisted migration efforts. For instance, Razgour et al. (2019) uncovered adaptive differences related to spatial variation in climate in two Mediterranean bat species (*Myotis escalerai* and *M. crypticus*) by analysing ddRAD data with GEA. Incorporating this climate‐adaptive potential into forecasts of range changes under climate change reduced projected range reductions, highlighting the importance of taking adaptive potential into consideration in climate change vulnerability predictions. The Figure shows this conceptual framework, reprinted from Razgour et al. (2019). Similarly, Bay et al. (2018) identified genomic variation associated with climate across the breeding range of yellow warblers (*Setophaga petechia*). They found that populations that will require the greatest shifts in allele frequencies at these adaptive loci to keep pace with climate change have already experienced the most severe population declines, suggesting that inability to adapt to a changing climate may already be causing declines.

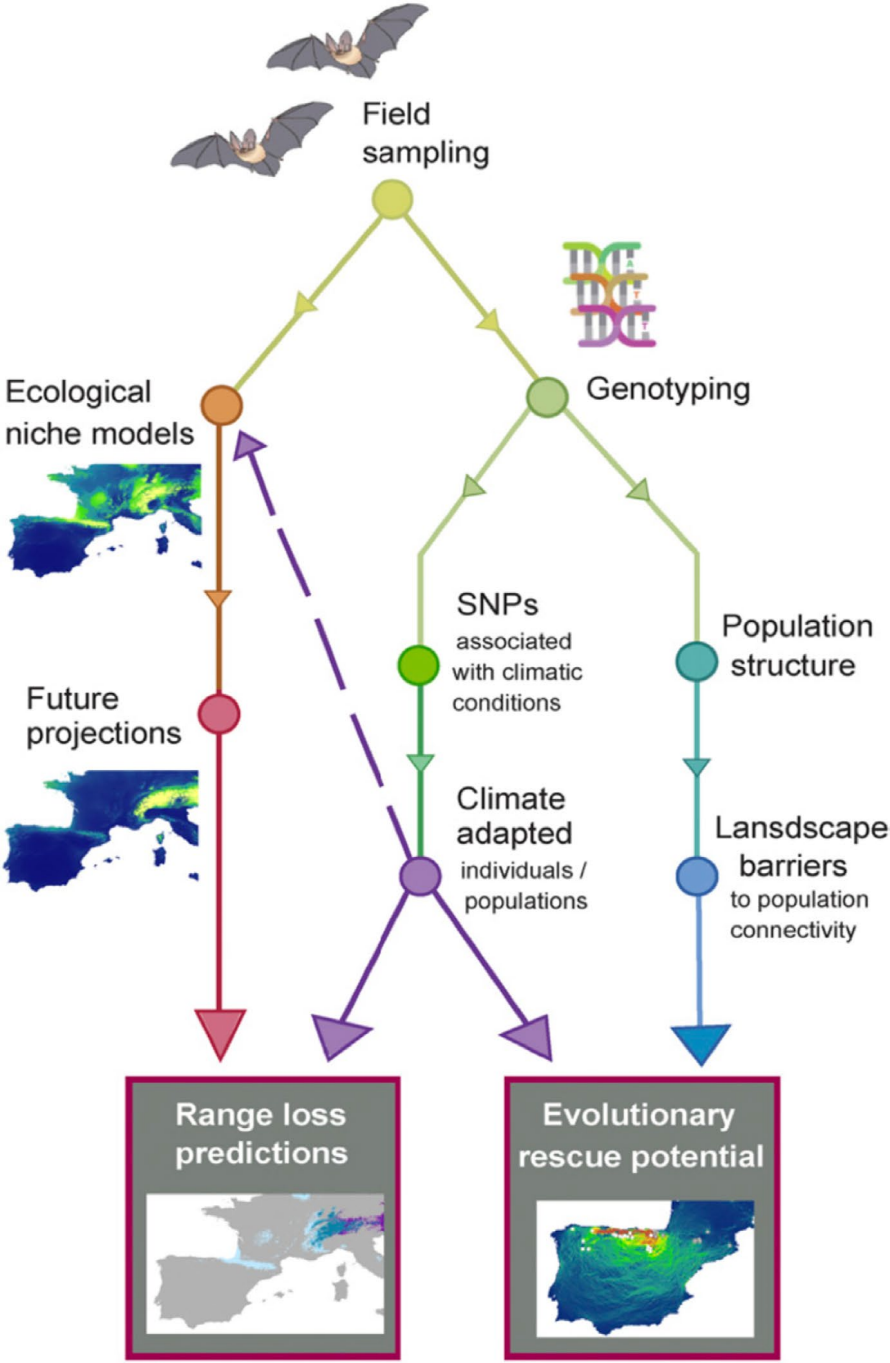

### 3.2 Identifying adaptive genetic variation

Adaptative variation in contemporary wildlife populations is often most evident as differentiation among populations or across a landscape where selective factors, such as interacting species or climate, are heterogeneous. One analytical framework for identifying loci under selection is outlier tests (Beaumont & Nichols, 1996). These tests allow detection of loci with “outlying” behaviour, such as unusually high or low *F*_ST_ values, potentially indicative of divergent or stabilizing selection, respectively. Although *F*_ST_ outlier tests have proved an important approach for identifying loci under selection, a number of factors ranging from recombination rate variation across the genome to demographic fluctuations can produce large variance in *F*_ST_ and related statistics. Several recent papers have cautioned that they can be subject to high type I error rates as a result (Hoban et al., 2016; Whitlock & Lotterhos, 2015). Genotype environment associations (GEAs) are another method for identifying loci under selection in a landscape genomics framework (Forester et al., 2018). The goal of GEAs is to identify loci that have allele frequencies that are associated with environmental gradients hypothesized a priori to drive local adaption (Rellstab et al., 2015). GEA analyses are more powerful than *F*_ST_ outlier tests because they make use of an additional source of data (Forester et al., 2018; De Mita et al., 2013), but they can only identify loci associated with the environmental gradients included as predictor variables in the analysis. Environmental variables also may be strongly correlated with each other and with geographical distance, making associations with individual variables difficult to detect.

Within populations, adaptive variation and genomic signatures of selection can be detected if samples are available over multiple generations (Gompert, 2015; Mathieson & McVean, 2013). This is possible for many wildlife species that have been the subject of long‐term studies, and also where museum specimens can be used as historical genetic samples (Dehasque et al., 2020). For example, Epstein et al. (2016) identified two genomic regions showing signatures of selection in response to an epidemic disease – devil facial tumour disease (DFTD) in Tasmanian devils (*Sarcophilus harrisii*) – by applying RADseq to samples collected both before and after the disease appeared in three independent populations that were the focus of long‐term field studies. Signatures of selection in this case are shifts in allele frequency and LD at specific genomic locations, and concordant signatures across populations are evidence for an adaptive response. Similarly, Bi et al. (2019) applied sequence capture methods to museum and contemporary samples from two chipmunk species (*Tamias* spp.) spanning a century and identified significant shifts in allele frequencies. Neither of these studies specifically included phenotypic data on potential adaptive traits; nonetheless, both identified specific candidate genes with known function that may affect fitness under changing selection regimes in natural populations.

A complementary approach to determine the genetic basis of adaptative variation in natural populations is genome‐wide association studies (GWAS) (e.g., Bérénos et al., 2015; Husby et al., 2015). The goal of GWAS is to identify loci and alleles underlying phenotypic variation by gathering large‐scale genomic and phenotypic data on a set of individuals. For instance, using some of the same long‐term Tasmanian devil population studies described above, Margres et al. (2018) used GWAS to identify loci associated with three DFTD‐related phenotypes and found that genetic factors explained a large proportion of the variance in infection status and survival after infection of female Tasmanian devils. This study used a hybrid RADseq and sequence capture approach and a pre designed panel of nearly 16,000 markers that included some candidate selected loci from Epstein et al. (2016). GWAS often require large sample sizes for sufficient statistical power (Kardos, Husby, et al., 2016), but this case illustrates how GWAS can be complementary to selection studies, providing a multi pronged population genomics approach to understand the genetic basis of adaptation in wildlife populations. All of these sources of data can be applied to predictive models of adaptation (Box 2) and to guide monitoring and genetic management of wildlife populations (discussed below).

### 3.3 Deleterious variation

In addition to identifying loci that can provide the capacity to adapt to environmental change or local conditions, population genomics can also reveal the genetic basis of reduced fitness in small populations. A central paradigm in conservation genetics is that genetic drift in small populations can cause inbreeding depression, reduce individual fitness, decrease population size and increase extinction probability, what has been referred to as the “extinction vortex” (Soulé & Mills, 1998). Deleterious alleles can rise to high frequency due to genetic drift, and mating between close relatives in a small population can increase the expression of recessive deleterious alleles in the homozygous state and reduce genome‐wide heterozygosity, reducing individual fitness. Identifying populations with low genetic variation, small effective population sizes and evidence of inbreeding depression is of paramount importance for the conservation of wildlife populations.

Population genomics provides tools to understand the genetic basis of reduced fitness in small wildlife populations and potentially address the issues through management actions. For example, Apennine brown bears (*Ursus arctos marsicanus*) comprise a small, isolated population in Italy. Benazzo et al. (2017) used WGS to discover that all variation was lost in the mitochondrial genome and parts of the nuclear genome, and several deleterious alleles were fixed, with predicted effects on physiology, development and behaviour. These analyses are possible with annotated reference genomes, on which regions of reduced variation can be mapped and the functional consequences of mutations in specific genes can be predicted (e.g., by analysing genomic data from island foxes [*Urocyon littoralis*] with the domestic dog [*Canis domesticus*] reference genome, Robinson et al., 2016; also see Box 1).

In addition to current population size, the demographic history of a population can have important and sometimes counter intuitive effects on population fitness. For instance, the long‐term effective population size is lower in a population that has been small for a long time, compared to one with a recent rapid decline. Nonetheless, the genetic or mutational load – the fitness cost of accumulated deleterious mutations – can be lower in the first case and more severe in the second, because strongly deleterious mutations can be purged during an extended period of small size with inbreeding (Robinson et al., 2018; van der Valk, Díez‐del‐Molino, et al., 2019; van der Valk, de Manuel, et al., 2019). In wildlife species, this means that reduced population fitness may be more of a problem in recent anthropogenic declines compared to populations that were small before human influence. Conversely, the genetic effects of a population bottleneck can linger even after the population has recovered demographically. Grossen et al. (2020) found that population bottlenecks in successfully reintroduced Alpine ibex populations (Figure 1) had purged highly deleterious mutations while allowing mildly deleterious ones to accumulate. As a result of all of these factors, there may often be little relationship between genetic diversity or genetic load and current population size, so that these genetic factors may not be reflected in conservation status assessments such as IUCN listing (Díez‐del‐Molino et al., 2018; van der Valk, de Manuel, et al., 2019).

## 4 INFORMING MANAGEMENT ACTIONS

Although application of population genomics to wildlife conservation and management has been slow to develop (Shafer et al., 2015), population genomics studies are already generating information that can help wildlife managers and conservation practitioners make difficult management decisions (Walters & Schwartz, 2020). We highlight specific examples of the application of population genomics to conservation and management of wildlife populations here.

### 4.1 Identifying population units

One of the most important first steps for managing populations is to identify and delineate the boundaries of intraspecific conservation units (CUs), such as evolutionarily significant units (ESUs) and management units (MUs). We define an ESU as a classification of populations that have substantial reproductive isolation and adaptive differences so that the population represents a significant evolutionary component of the species (Funk et al., 2012). An MU is a local population that is managed as a separate unit because of its demographic independence. An ESU may contain multiple MUs. CUs may be further defined on the basis of specific adaptive variation (e.g., Prince et al., 2017). These definitions implicitly rely on multiple concepts of connectivity among populations, including demographic and multiple aspects of genetic connectivity, which may be substantially different; for instance, the level of migration needed to avoid inbreeding depression and loss of adaptive genetic variation may be much lower than that needed to maintain demographic connectivity and directly increase population size through immigration (Lowe & Allendorf, 2010).

**Figure 3:**
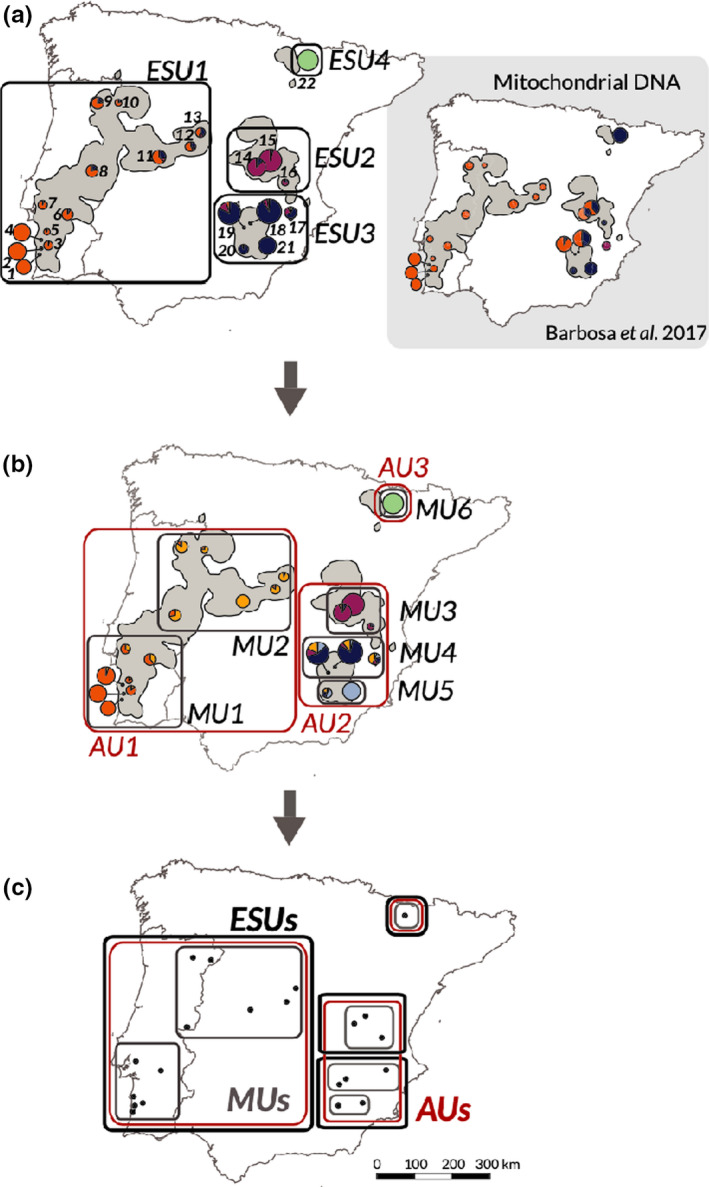
Designation of conservation units in Cabrera voles (*Microtus cabrerae*) across the Iberian Peninsula. Genome‐wide variation estimated from reduced representation sequencing provides greater resolution of evolutionarily significant units (ESUs) than previous microsatellite results. Neutral and adaptive variation facilitated delineation of management units (MUs) and adaptive units (AUs), respectively. Reproduced from Barbosa et al. (2018)

Population genomics tools can be applied to estimate multiple aspects of population structure and connectivity, and in some cases have led to changes in management. The population genomics work of Andrews, Nichols, et al. (2018) revealed that one population (of canary rockfish, *Sebastes pinniger*) listed under the U.S. ESA did not actually merit listing as a discrete population, while a second (yelloweye rockfish, *S. ruberrimus*) harboured previously unknown genetic differentiation. Genomics studies have more power than previous microsatellite studies to quantify overall (genome‐wide) population differentiation; for instance, McCartney‐Melstad et al. (2018) applied RADseq data to the declining foothill yellow‐legged frog (*Rana boylii*) and found five extremely differentiated clades that can serve as management units for this species of conservation concern. Barbosa et al. (2018) used reduced representation sequencing data following the framework of Funk et al. (2012) to delineate CUs in Cabrera voles (*Microtus cabrerae*): ESUs on the basis of overall differentiation, MUs on the basis of differentiation at neutral loci and adaptive units (AUs) on the basis of outlier loci (Figure 3). Previous results from environmental niche modelling and landscape genetics connectivity analysis are also informative for designing strategies in this species (Barbosa et al., 2018). Once populations are delineated, the genomic data can also provide high‐throughput genotyping panels for assigning individuals to populations, and adaptive loci may be particularly useful for this effort (Larson et al., 2014). For example, in anadromous fish species in which multiple breeding populations mix during the oceanic phase where they may be subject to harvest, breeding populations can be distinguished on the basis of some combination of neutral and adaptive genetic markers (Waples et al., 2020).

### 4.2 Genetic monitoring

Genetic monitoring of natural populations has played an important role in conservation, including both monitoring of genetic diversity and using genetic tools to monitor other aspects such as population size or hybridization. The advent of population genomics presents new opportunities for improving the utility of genetic monitoring for wildlife (Flanagan et al., 2018; Hunter et al., 2018; Leroy et al., 2018; Mimura et al., 2017). First, as described above, genomics tools can be used to rapidly design a relatively small set of genetic markers that can be genotyped efficiently across many individuals, often using minimally invasive sampling (Carroll et al., 2018). These marker panels can be designed for specific goals, such as estimating population size or detecting hybridization. More importantly, population genomics tools also allow monitoring of allele frequency changes at adaptive loci. Monitoring changes at these loci can track changes in adaptive potential as a result of environmental change or management actions, such as assisted migration or genetic rescue, so that management strategies can be continually updated (Flanagan et al., 2018). Monitoring of deleterious variants, such as those that cause inbreeding depression, could also be informative to detect genomic erosion in small populations (Leroy et al., 2018). If monitoring reveals that genetic problems are accumulating, or that a population is not showing evidence of an adaptive response to environmental stressors, it would suggest more active management strategies. Conversely, monitoring genetic variation at adaptive loci can inform managers on whether evolutionary rescue is possible. For instance, in the case of Tasmanian devils and their transmissible cancer described above, population genomics studies have revealed loci associated with a rapid response to selection and with particular disease‐related traits. Genetic monitoring panels could specifically assay these loci to ensure that sufficient variation exists, both in natural and in captive populations (Hohenlohe, McCallum, et al., 2019).

### 4.3 Assisted gene flow, genetic rescue and translocations

As wildlife populations become increasingly isolated in a fragmented world, managers are faced with the decision of whether or not to restore gene flow by moving individuals between populations to rescue them from population declines caused by the loss of genetic variation. Genetic rescue is an increase in population fitness and decrease in extinction probability caused by gene flow (Bell et al., 2019; Tallmon et al., 2004; Whiteley et al., 2015). Genetic rescue may occur by reducing inbreeding depression via masking deleterious alleles expressed in the homozygous state, or by infusing additive genetic variation on which selection can act so that populations can adapt to changing environments (evolutionary rescue). Fitzpatrick and Funk (2019) outline a variety of ways in which population genomics can help managers with decisions regarding genetic rescue. First, genomics tools can help identify populations suffering from low genetic variation and inbreeding depression, as outlined above, and map regions of low variation across the genome (Box 1). Second, genomics can help identify the best potential source populations that are not too adaptively divergent from the target recipient population. A fine‐scale genomic view could potentially identify source populations that best reduce genomic regions of homozygosity while minimizing disruption of local adaptation. Finally, if and when genetic rescue is implemented, genomic data can be used to monitor changes in genetic ancestry across loci and the relative fitness of immigrants, residents and hybrids to test whether gene flow is increasing fitness as desired (Miller et al., 2012).

A number of genetic rescue attempts have been conducted in wildlife populations, and some general trends are emerging (Bell et al., 2019). A risk of genetic rescue is outbreeding depression – reduced fitness when assisted migration comes from a divergently adapted source population. Some authors have suggested that outbreeding depression may be a low risk in most cases (Frankham, 2015; Chen, 2019; Fitzpatrick et al., 2020). In many wildlife species, the problems of small populations and inbreeding depression may be the fairly recent effect of human‐caused fragmentation; in this case, these populations would not be expected to be highly divergent adaptively, and assisted migration is more likely to be appropriate (Ralls et al., 2018). In contrast, attempts at genetic rescue could impede ongoing evolutionary rescue if populations are already rapidly evolving to a novel environmental condition, such as a disease (Hohenlohe, McCallum, et al., 2019). In this case, population genomics tools can identify the pace and genetic nature of this adaptation and inform management decisions.

### 4.4 Managing for specific genetic variants

For threatened and declining populations, a major concern is that adaptive alleles might be lost by environmental stressors caused by humans. Prince et al. (2017) made the surprising discovery that variation in a major life history trait in salmon – migration timing – is underpinned by the same single locus across multiple populations in two different species, Chinook salmon (*Oncorhynchus tshawytscha*) and steelhead (*O. mykiss*). Thompson et al. (2019) then went on to test the effects of a recently constructed dam on adaptive potential at this locus, given that the dam selects against the spring‐run phenotype because fish with this phenotype historically spawned upstream of the dam. They found a dramatic reduction in the frequency of the spring‐run phenotype and allele underlying this phenotype. Simulations suggest that the dam could lead to the complete loss of this allele in the near future. This situation highlights a conundrum: in general, it may be inadvisable to manage populations on the basis of a single allelic variant, because it could neglect important factors across the rest of the genome. In this case, however, a substantial ecological role and associated phenotypes could be lost with the loss of this single allele.

Most genetic variation that is important to management is likely to be polygenic, although there may be wide variation among populations and taxa. The number of loci affecting fitness or adaptive capacity depends on the population history, and whether large‐effect or small‐effect allelic variation plays a bigger role in either adaptive or deleterious variation (Grossen et al., 2020). Population genomics tools are able to identify dozens to hundreds of candidate loci associated with a trait or with fitness, and lead to high‐throughput genotyping assays that could target these loci (perhaps in combination with others). Most studies do not have the statistical power to resolve the specific effects of each locus or even identify them with high confidence (Hoban et al., 2016), and this will remain an unavoidable problem with the sample sizes available in many wildlife populations (Margres et al., 2018). Thus, active management to favour particular alleles could not be supported in these cases. However, management strategies with genetic monitoring could be designed to maintain variation at these loci, for instance in captive populations and with the additional goal of maintaining variation genome‐wide (Hogg et al., 2019), so that adaptive evolution is possible in the wild.

### 4.5 Ex situ management

Many wildlife species are kept in captivity, and some of these are either extinct in the wild or limited to populations smaller than those in captivity, so that the captive populations represent the majority of genetic variation in the species (e.g., Humble et al., 2020). These are often subject to intensive genetic management and some degree of controlled breeding, and genomics tools can be applied in multiple ways (Brandies et al., 2019). For instance, methods to estimate demographic history, source population or admixture can reveal much about captive individuals. Genomics tools can rapidly provide marker sets for efficient genotyping. Even when pedigree relationships are completely known, genomic data can provide more precise estimates of actual genetic relatedness, inbreeding and the proportion of the genome that is identical by descent (Kardos et al., 2015; Box 2). Controlled breeding can be precisely designed to maximize genome‐wide diversity, to maintain genetic distinctiveness of source populations, or potentially to manage for variation at particular loci as described above. Selection for traits that are favoured in captivity but maladaptive in the wild is a major problem for captive populations, and genetic monitoring could focus on specific loci associated with adaptation to captivity.

## 5 IMPROVING CONNECTIONS BETWEEN POPULATION GENOMICS AND CONSERVATION

We have several different recommendations to improve translating the power of population genomics research into better wildlife conservation and management decisions. Although population genomics clearly provides unprecedented power to peer into the genomes of wildlife species, a gap still remains between population genomics research and application to conservation practice (Garner et al., 2016; Shafer et al., 2015).

Our first recommendation is for population genomicists to develop professional relationships with managers and conservation practitioners. The old model of conducting research, writing a paper on the results with a “conservation recommendations” section at the end, and then expecting managers to find and use the research has been shown to be ineffective at impacting management decisions. Fabian et al. (2019) surveyed Swiss professionals in nature conservation and found that experience‐based sources (e.g., personal exchange with colleagues and experts) are more important than evidence‐based sources (e.g., printed products and journals). Articles in scientific journals were almost never consulted by conservation practitioners. Given that conservation professionals have limited time to read scientific articles and keep up with the rapid pace of advancement in fields such as population genomics, it is essential for scientists to build relationships and communicate directly with managers and conservation practitioners if they want their science to improve conservation management and policy. Holderegger et al. (2019) describe multiple frameworks, such as workshops, modes of communication and joint projects, that can facilitate connections between researchers and practitioners.

A second recommendation is to let conservation and management questions guide research. Often, a study or results that a researcher thinks are useful for conservation may not be what a manager needs to know to make decisions that affect wildlife species. Ultimately, research results can only guide conservation if they have bearing on management decisions. Thus, researchers first need to know what decisions managers face and what management actions are within the realm of possibility, and this communication should happen early in the research process (Holderegger et al., 2019). Only then can researchers know what questions managers need answered to help them decide the best management option. Building relationships with managers, as above, is extremely helpful for learning about the problems and issues that managers and conservation practitioners are faced with, where information gaps exist, and how research can fill these information gaps. Relationships with managers will also provide opportunities for researchers to communicate the types of questions that population genomics can and cannot help answer.

Another recommendation for improving the translation of population genomics into improved wildlife conservation and management is training for both aspiring population genomics students and conservation practitioners, ideally together to foster direct interaction between these groups. Population genomics workshops, for example, not only provide technical training in the ever‐expanding field of genomics; they can also provide opportunities for conservation practitioners to gain exposure to the field to give them a better appreciation of the capacity of population genomics, the steps involved, and how to apply it to the species they manage and the questions they face. Fortunately, several genomics workshops now provide venues to discuss the latest developments in population and conservation genomics, such as the annual Population and Conservation Genomics workshop at the International Plant and Animal Genomes Conference (https://intlpag.org), and hands‐on training in population genomic analysis, including the ConGen workshop at the University of Montana's Flathead Lake Biological station (http://www.umt.edu/sell/cps/congen2019/), the Genomics of Disease in Wildlife workshop at Colorado State University (https://gdwworkshop.colostate.edu/), and a variety of workshops given across Europe by the G‐BIKE (Genomic Biodiversity Knowledge for Resilient Ecosystems) programme (https://sites.google.com/fmach.it/g‐bike‐genetics‐eu/home).

A final recommendation is for the population genomics community to continue streamlining and standardizing bioinformatics tools and population genomics analyses. Many bioinformatic pipelines and population genomics analyses require fairly advanced computer and programming skills, in addition to understanding of population genetics concepts. These factors can act as a barrier to entering the “genomics world” for many students, scientists and conservation practitioners, given the relative ease of producing genomic data. Bioinformatics tools and population genomics analyses need to be developed that are more broadly accessible. Moreover, bioinformatics pipelines and guidelines for best practices have not yet been standardized. Fortunately, significant progress is being made in the development of more user‐friendly programs and clear guidelines for collecting and applying genomics to wildlife biology and management (Gomez‐Sanchez & Schlötterer, 2018; Gruber et al., 2018; Ravindran et al., 2019).

## 6 CONCLUSIONS AND FUTURE PROSPECTS

Even in the relatively short time (~10 years) since genomic data have been applied to population genetic questions in nonmodel organisms, population genomics has already helped answer a wide variety of questions in the biology of wildlife species. There has been a relatively slow uptake of population genomics results in influencing policy decisions and wildlife management actions (Shafer et al., 2015), with a number of factors contributing to significant time lags: researchers learning how to apply population genomics in wildlife species, studies being completed through publication of results, communicating results and interpretation of genomic data to conservation practitioners, integrating genomic results into the many sources of information that influence policy decisions or conservation actions, etc. Nonetheless, a decade on, examples of direct connections between population genomics research and wildlife conservation actions are now rapidly accumulating (Walters & Schwartz, 2020). A remaining question, however, is whether population genomics can help stem the tide of cataclysmic biodiversity declines given the accelerating urgency of the problems.

Population genomics research is by nature intensive and focused on one or a few species. It has, therefore, been applied to wildlife species that are high‐profile or of significant economic interest, such as captive populations or salmonid fish (Waples et al., 2020), although the decreasing costs of genomic studies and proliferation of resources such as reference genome assemblies have allowed these techniques to spread across taxa, and this trend will continue. Future directions include expanding the “omics” toolkit to include transcriptomics, epigenomics or proteomics, which may improve our understanding of adaptive capacity in wildlife populations and the role of gene expression, epigenetics and phenotypic plasticity in population fitness. There may also be a role for genetic engineering techniques in wildlife, such as gene therapy or gene drive approaches to cause alleles to spread in a population (Breed et al., 2019; Rode et al., 2019). In species that suffer from a well‐understood, relatively simple genetic problem, it could be conceivable to use a “rescue drive” – an attempt to spread a favoured allele into a population to increase fitness (Rode et al., 2019). However, this approach carries numerous poorly understood risks, including the pitfalls associated with focusing management on a narrow set of genetic factors (Kardos & Shafer, 2018). Another approach is to use gene drive techniques to control or eradicate invasive species that negatively affect native wildlife (Rode et al., 2019). While invasive species can often require active management, and some level of risk may be acceptable compared to taking no action, the risks of such eradication or suppression drives are still poorly known.

A future need in conservation is to understand how population genomics tools can be applied more broadly beyond single focal species, for instance at the ecosystem level (Breed et al., 2019). One avenue is metagenomics or metabarcoding approaches, where genetic samples include multiple species, for instance with eDNA (Goldberg & Parsley, 2020). Population genomics focused on species that are central to ecosystem interactions may also reveal the community effects of genomic diversity (Hand et al., 2015). These may often be plants, such as the dominant tree species in a forest ecosystem in which many other species are affected by its genetics, and genomics tools can be important for seed sourcing in restoration efforts (Breed et al., 2019). In other cases, wildlife species may play a similar role.

The field of population genomics continues to change rapidly, with technological and analytical advances expanding the tools that are available in wildlife biology at the same time as the need for conservation knowledge and action becomes more urgent. While it may be very difficult to keep up to date with all of the changes, it is critical for both researchers and wildlife professionals to maintain a broad understanding of the population genomics tools that are available and to foster communication between wildlife scientists and practitioners.

## AUTHOR CONTRIBUTIONS

All authors contributed to writing the manuscript.

## Data Availability

No new data were generated or analysed as part of this review article.
